# Modulatory effects of bioactive natural compounds on pruritic pathways: mechanistic basis and therapeutic prospects

**DOI:** 10.1007/s10787-025-01999-1

**Published:** 2025-10-30

**Authors:** Wagih H. Marcus, Hassan A. Ruby, Amira E. Awwad, Mahrous H. Mahrous, Ebraheem Abouelwafa, Mohamed A. S. Badawy, Riham A. El-Shiekh, Eman S. Zaki, Mahmoud Abdelmouti Mahmoud

**Affiliations:** 1 Department of Pharmacology and Toxicology, Faculty of Pharmacy, Cairo University, Cairo, 11562 Egypt; 2 Pharmacognosy Department, Faculty of Pharmacy, October 6 University, Sixth of October City, 12585 Egypt; 3 Department of Pharmacognosy, Faculty of Pharmacy, Delta University for Science and Technology, Dakhliya, Egypt; 4 Pharmacognosy Department, Faculty of Pharmacy, Merit University (MUE), Sohag, 82755 Egypt; 5 Department of Pharmaceutical Chemistry, Faculty of Pharmacy, Merit University (MUE), Sohag, 82755 Egypt; 6 Pharmacognosy Department, College of Pharmacy, Cairo University, Cairo, 11562 Egypt; 7 Department of Pharmacology, Faculty of Pharmacy, Heliopolis University, Cairo, 11536 Egypt; 8 Department of Biochemistry, Faculty of Pharmacy, Cairo University, Cairo, 11562 Egypt

**Keywords:** Medicinal plants; Pruritus; Complementary medicine

## Abstract

Chronic pruritus, characterized by persistent itching, is a significant health issue that adversely affects individuals’ quality of life, particularly in palliative care environments. Conventional treatments frequently do not yield sufficient relief and may lead to undesirable side effects, which has spurred interest in exploring alternative therapeutic options. A thorough literature search was conducted across several databases such as PubMed, Web of Science, Scopus, and EKB, with an emphasis on studies involving in vitro, animal models, and clinical trials utilizing terms like “plant,” “extract,” and “pruritus.” All studies were considered regardless of their publication date and were limited to English-language articles. This review highlights the promise of various medicinal plants, including chamomile, *Aloe vera*, calendula, curcumin, lavender, licorice, and peppermint, as adjunctive therapies for pruritus. These plants possess a range of pharmacological properties, such as anti-inflammatory, antioxidant, and skin-soothing effects, which are relevant to the complex nature of pruritus. Their favorable safety profiles and natural origins enhance their appeal in holistic and patient-centered care models.

## Introduction

Pruritus or itching is the most common skin disorder, characterized by an irritating sensation that evokes a scratch. It manifests either acute or chronic (> 6 weeks). It impacts the daily activities, sleep patterns, and mood of patients (Yosipovitch et al. 2018a; Anzelc and Burkhart 2020). Almost 40% of the population suffers from itching with a high prevalence in women and the elderly (> 65) (Yosipovitch et al. 2024). Pruritus is caused by hypersensitivity or a symptom of various diseases, such as skin conditions (eczema, xerosis, skin cancer), systemic conditions (liver insufficiency, thyroid dysfunction, HIV). Furthermore, it may arise from neurogenic diseases (shingles, and diabetes mellitus) or psychogenic problems (generalized anxiety disorder) (Shevchenko et al. 2018). Therefore, it requires an evaluation via assessment of medical history, biopsies, laboratory tests, and imaging. Also, the intensity of itching should be frequently measured using validated scales (0–10) to monitor treatment efficacy (Rupert 2022). The mechanism of pruritus encompasses a complicated mingling across many receptors, ion channels, and immune mediators. The onset of the itching signal might vary, and pruritus can be either acute or chronic (Metz and Ständer 2010). In this circumstance, afferent nerve fibers and skin cells interact in a complicated way (Szöllősi et al. 2022). Historical myths that itch is communicated by pain fibers or symbolizes low-intensity pain have been debunked, as itch and pain are now established as independent sensory modalities with separate brain pathways, despite occasional interactions(Najafi et al. 2021). The histamine-dependent and independent groups of itchy fibers are involved via different cascades (Brennan 2016). Notably, 90% of itchy fibers are histamine independent, whereas 10% are histamine dependent, considering the fact that their relationship is complicated (Davidson et al. 2007).

Pruritus pathophysiology commences with the excitation of cutaneous sensory neurons by pruritogens, principally via activation of G protein-coupled receptors (GPCRs) expressed on peripheral nerve terminals. This GPCR engagement elicits downstream signaling cascades culminating in the opening of specific ion channels, notably Transient Receptor Potential Vanilloid (TRPV1) and (TRPV3). Moreover, Transient Receptor Potential Ankyrin 1 (TRPA1), proteases activated receptors (PARs) (Shevchenko et al. 2018). Consequent neuronal depolarization generates action potentials that propagate the pruritic signal centripetally. Acute pruritus is predominantly mediated by histamine, released from resident immune cells such as mast cells. Histamine binding to neuronal histamine H1 and H4 receptors directly activates pruriceptive neurons, inducing concomitant inflammatory responses including erythema, edema, and inflammation (Green and Dong 2016). Conversely, chronic pruritus frequently involves non-histaminergic mechanisms, driven by cytokines such as interleukin-4 (IL-4), interleukin-13 (IL-13), and particularly interleukin-31 (IL-31). These cytokines directly stimulate sensory neurons through pathways independent of histamine receptor activation (Yosipovitch et al. 2018a). Furthermore, pruritus severity, irrespective of acute or chronic etiology, is potentiated by additional mediators, including serotonin and interleukin-6 (IL-6) (Konda et al. 2015).

The management of acute and chronic pruritus is based on non-pharmacological and pharmacological approaches. Non-pharmacological methods include acupuncture (Tang et al. 2022), phototherapy aided by UV radiations (Shevchenko et al. 2018) and therapeutic bathing regimens (Fourzali et al. 2019). While Pharmacological management of pruritus encompasses topical treatments for acute cases and systemic oral therapies for chronic ones. Topical corticosteroids, the first treatment line, although they reduce histaminergic itch yet the prolonged use causes skin atrophy and thinning (Fourzali et al. 2019). However, the immuno-modulators as topical calcineurin inhibitors reduce Th1 cytokines, but they cause an initial stinging (Yosipovitch et al. 2018a). Systemic oral treatments of chronic pruritus either act on the nervous system or the immune system. Despite the histamine receptors (H1/H2) antagonists are effective in the treatment of chronic urticaria, they aren’t useful in most chronic itching conditions. Furthermore, they have anticholinergic side effects such as dry mouth, double vision, and urinary discomfort and may increase the risk for Alzheimer’s disease particularly in the elderly (Fourzali et al. 2019). Additionally, chronic itching may be treated with other systemic therapies, such as anticonvulsants and antidepressants, but are associated with varied side effects and difficulty of dose control (Reszke et al. 2019). For these limitations of synthetic medications, the risk of drug interactions and hypersensitivity occurrence there is a high demand for exploring alternative and natural treatment approaches, especially in chronic cases with unknown reasons. For instance, topical treatment with mastic ointment (resin from *Pistacia lentiscus*) decreases cytokine production (Kishimoto et al. 2021). Capsaicin (an alkaloid obtained from *Capsicum* species) act on TRPV1, PARs and other important neurotransmitter of sensory modalities (Parvizi et al. 2022). Patients applying topical menthol oil achieved temporary improvement in itching through activation of TRPM8 (transient receptor potential melastatin 8) receptors owing to its cooling effect (Yosipovitch et al. 2018a). Citrusinine-II, a natural acridone alkaloid from *Atalantia monophylla*, is a potent and selective antagonist of the TRPV3 channel (Han et al. 2021).

This review aims to explore the current understanding of pruritus and its underlying mechanisms, detect the limitations of existing treatments. In addition, emphasize the potential of plants, their extracts and natural remedies as an alternative safe innovative solution for itch management. Furthermore, focus on the efficacy and safety of plant-based interventions to provide a promising perspective on advancing pruritus treatment strategies. Further research is essential to optimize their therapeutic potential and ensure safe, effective application.

### Physiological basis of pruritus

Pruritus, commonly known as itching, is an unpleasant sensation that causes the urge to scratch. It can be caused by different factors depending on the disease. For example, in eczema, it might be due to inflammation, while in liver disease, it could be due to bile salts accumulating in the skin (Ikoma et al. 2006). Despite the different causes, the way our body perceives itching is generally the same across various conditions. Substances that cause itching, known as pruritogens, are produced in the skin. These could be histamines, cytokines, or other chemicals. These mediators bind specific receptors on peripheral sensory nerve fibers distributed throughout the skin. Once bound, they trigger signals that travel along these nerve fibers. These signals are relayed to the dorsal horn of the spinal cord, which acts as a relay station for sensory information. Finally, the signals are sent to the brain, where they are perceived as the sensation of itching (Irie and Kabashima 2021).

### Cross-talking between skin cells and sensory nerve fibers

According to their initiation and propagation, pruritus or itch can be classified into two types: histamine-dependent and histamine-independent pruritus (Irie and Kabashima 2021).

1. **Histamine-dependent pruritus**: This type of itch is caused by the release of histamine from mast cells and basophils, typically in response to allergens or other triggers. It is often associated with acute itch, such as that caused by insect bites or allergic reactions, and usually responds well to antihistamine treatments.
2. **Histamine-independent pruritus**: This type of itch involves mediators and pathways that do not rely on histamine, including cytokines, proteases, and neuropeptides. Histamine-independent pruritus is more common in chronic itch conditions such as neuropathic itch or atopic dermatitis and does not usually respond to antihistamines. Other treatments targeting different pathways may be necessary for relief.

**Fig. 1:**
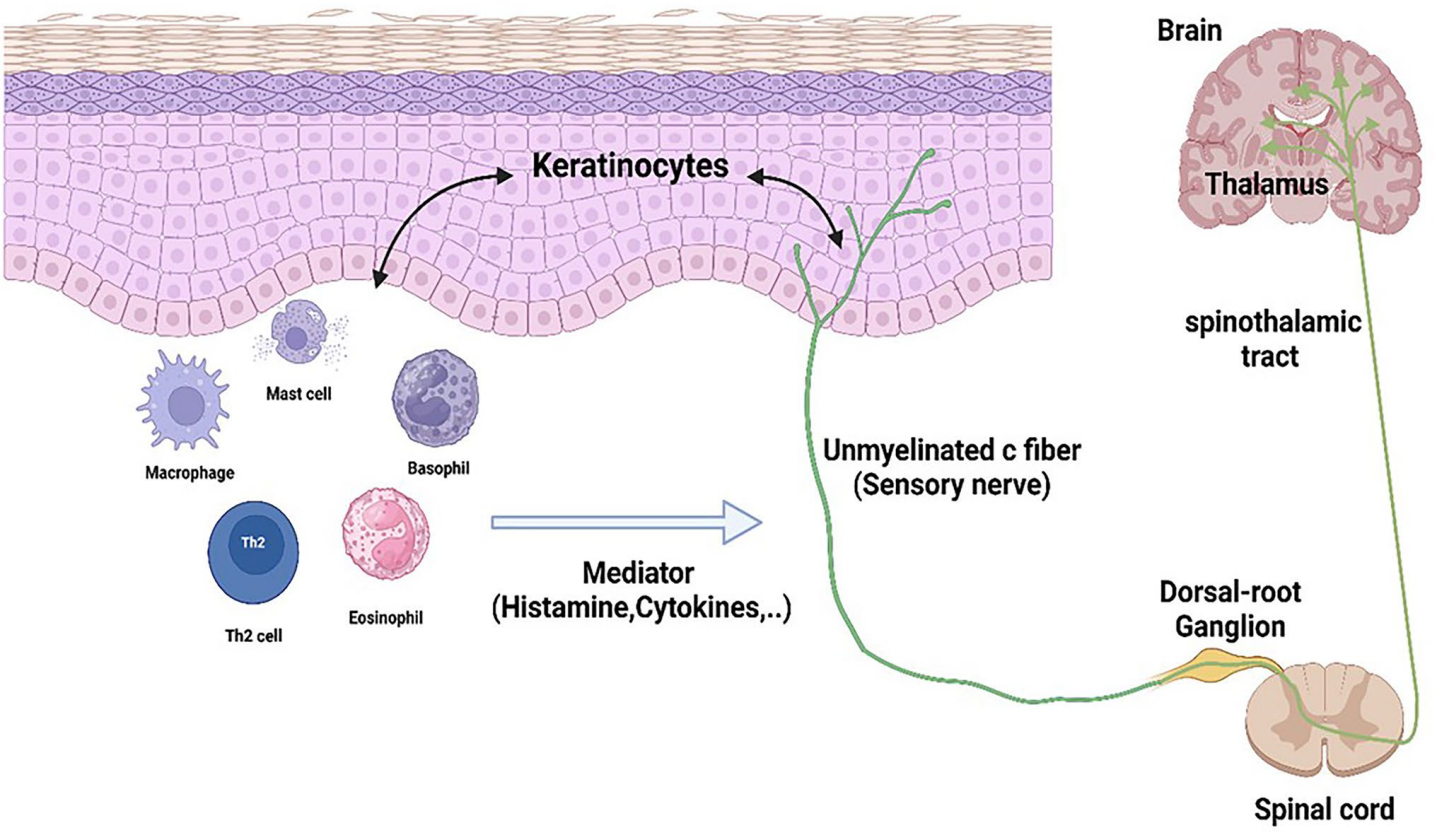
Interaction between immune cells, keratinocytes and neural components in the itch Signaling pathway

Many mediators, including histamine, cytokines, neuropeptides, protease and their receptors are involved in pruritus (Table 1), and these are produced by different types of immune cells, peripheral sensory nerves, and keratinocytes (Fig. 1).

**Table 1:** Main itch signaling molecules and their receptors

Class	Molecules	Receptors
Amines	Histamine serotonin	H1/H4 receptors 5-HT2 receptor
Neuropeptide	Substance p CGRP	Neurokinin 1 receptor CALCRL
Cytokines	IL-31 IL-4/13 TSLP	IL-31R/ oncostatin M receptor IL-4Rα TSLP receptor
Protease	Kallikrein Tryptase Cathepsin S	Protease activated receptor 2 (PAR2)

### Histamine

**Fig. 2:**
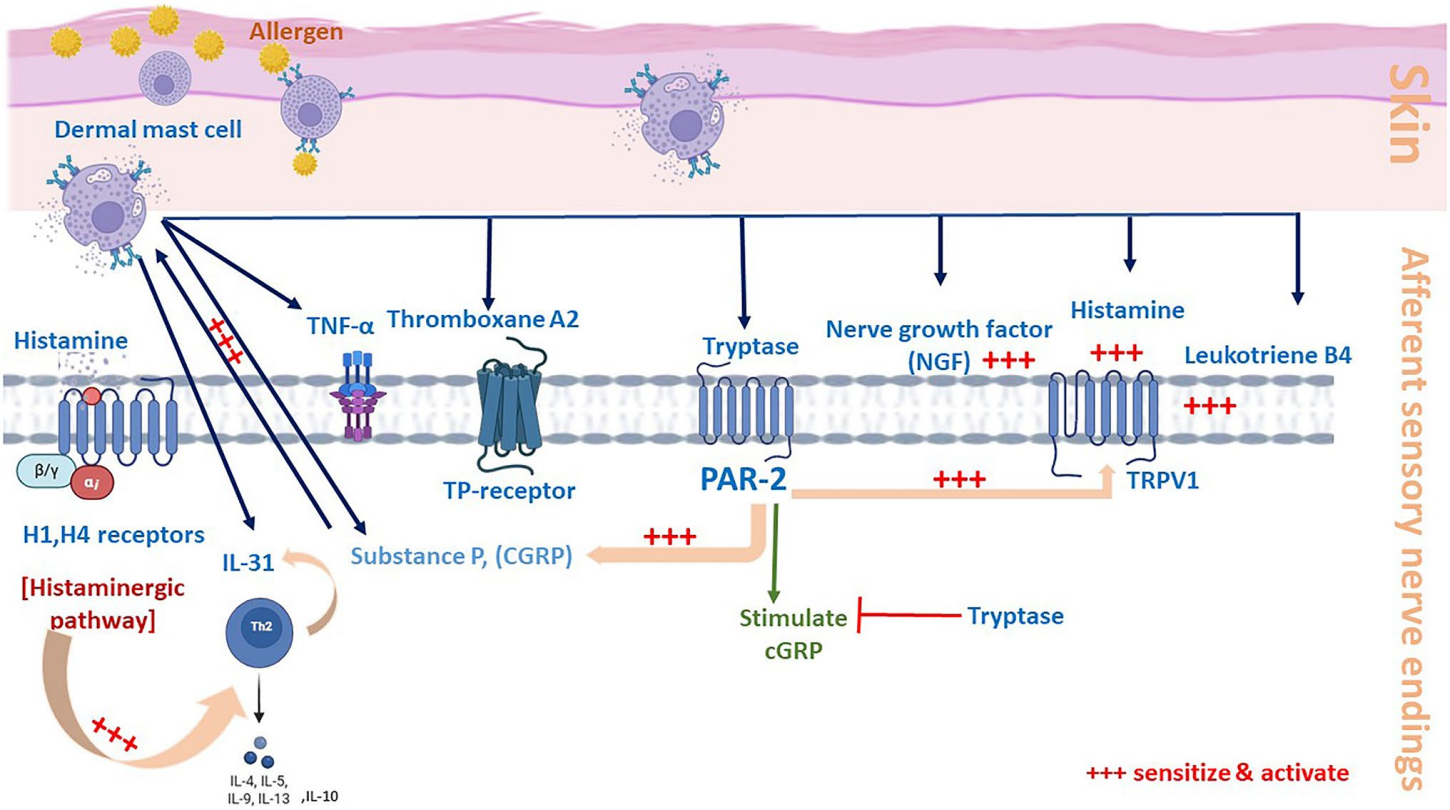
Skin cells, receptors, mediators and sensory nerve fibers crosstalk

Histamine is released from mast cells, basophil (Hashimoto et al. 2019), and keratinocytes (Shimizu et al. 2015), triggered by allergens, physical injury, or certain chemicals. When an allergen enters the body, it binds to immunoglobulin E (IgE) antibodies on the surface of mast cells and basophils, leading to the release of histamine. Once released, histamine binds to H1 and H4 receptors on sensory nerve endings in the skin and activates transient receptor TRPV1 through phospholipase A_2_ and lipoxygenase, or G_q/11_-PLCβ3 expression (Kittaka and Tominaga 2017). The activation of sensory nerves not only transmits the signal of itch to the brain but also leads to the release of neuropeptides like substance P and calcitonin gene-related peptide (CGRP), which exacerbate pruritus and cause inflammation (Schmelz and Petersen 2001). Histamine plays a multifaceted role in the amplification of pruritic and inflammatory responses. It enhances the secretion of Th2 cytokines, including IL-5, IL-4, IL-10, and IL-13, which are key mediators in allergic and inflammatory processes. Additionally, histamine activates mast cells, prompting the release of cytokines and chemokines (Thangam et al. 2018) (Fig. 2).

### Cytokines

Cytokines play a crucial role in the development and persistence of pruritus, functioning as signaling molecules that mediate and regulate immune responses, and interact with sensory nerves in the skin. This interaction bridges the immune and nervous systems, amplifying the sensation of itch. These molecules, including interleukins IL-31, IL-4, IL-13, and tumor necrosis factor-alpha (TNF-α) and thymic stromal lymphopoietin (TSLP). Moreover, this interplay of immune cells and these inflammatory mediators contributes to the inflammatory processes and neuro-immune interactions that underline various chronic pruritic conditions (Ong and Leung 2006; Yu et al. 2019).

For instance, IL-31, secreted by T-helper type 2 (Th2) cells, binds to its receptor on sensory neurons, triggering the JAK/STAT pathway and increasing peripheral nerve sensitivity to itch stimuli (Tsiogka et al. 2022). Meanwhile, TNF-α promotes vascular permeability and immune cell recruitment, such as mast cells and eosinophils, which release pruritogens like histamine, amplifying inflammation and itching (Lukacs et al. 1995; Aveleira et al. 2010) (Fig. 2).

Interleukin-31 (IL-31) is produced by different types of cells, including Th2 cells, mast cells, macrophages and eosinophils (Gibbs et al. 2019). IL-31 binds to IL-31 receptor A and oncostatin M receptor (OSMR) on the sensory nerve and activates TRPV1 and TRPA1 (Cevikbas et al. 2014), which transmits the signal of itch to the brain. Epithelial cells and keratinocytes express IL-31RA and OSMR. Upon activation by IL-31, these cells secrete various chemokines and inflammatory mediators, which play a role in the pathogenesis of pruritus (Dillon et al. 2004; Sonkoly et al. 2006).

IL-4 and IL-13, produced by Th2 cells and basophils play a role in chronic itch by binding to IL-4 receptor alpha (IL-4Rα) on these sensory nerve fiber (Yosipovitch et al. 2018b). IL-4 and IL-13 also impair the epidermal barrier by downregulating the expression of key proteins such as filaggrin (FLG), loricrin (LOR), and involucrin (IVL), increasing skin sensitivity and itchiness (Furue 2020).

**Fig. 3:**
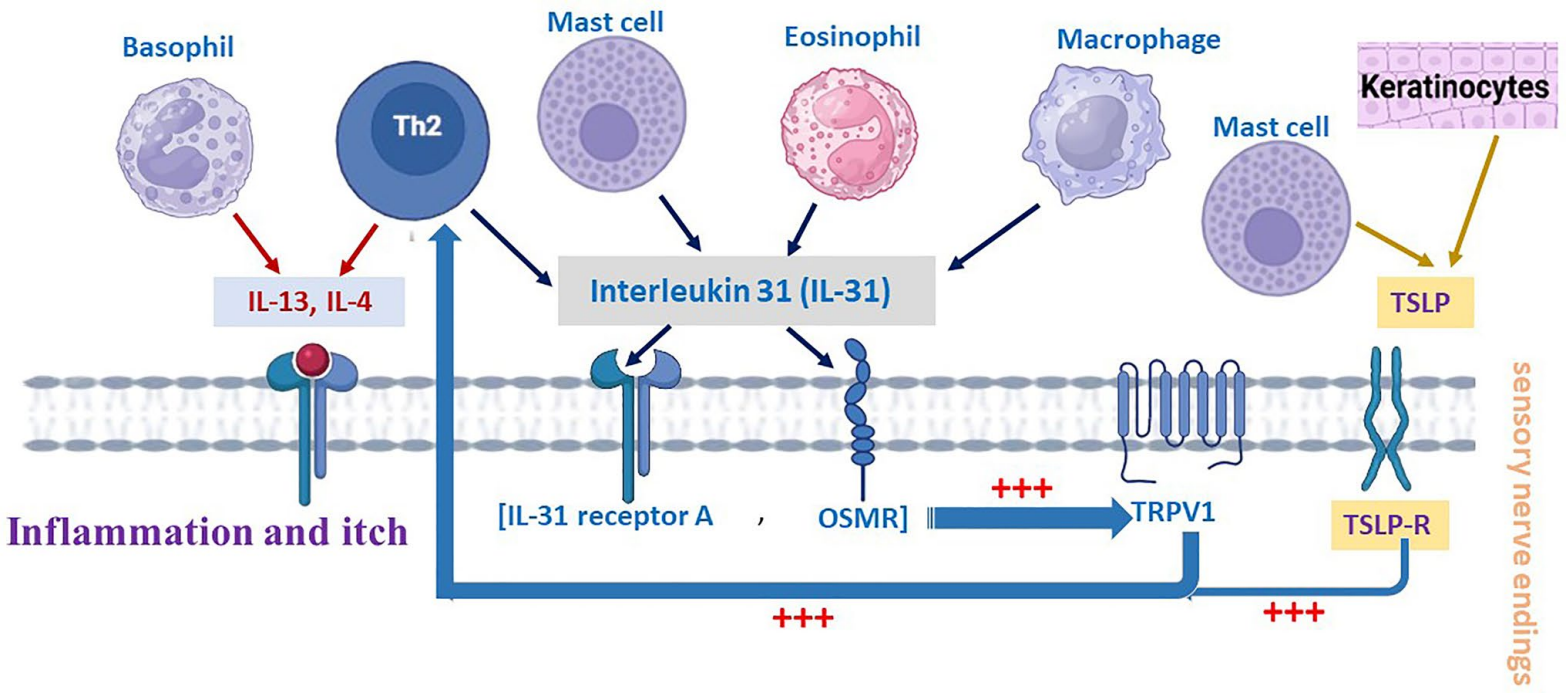
Cytokines critical role in pruritus

Thymic stromal lymphopoietin (TSLP) produced by keratinocytes and mast cells, contributes to pruritus by acting on the TSLP receptor on the sensory nerve fiber and activating TRPA1 (Wilson et al. 2013). TSLP promotes the differentiation and activation of Th2 cells, which release cytokines that contribute to inflammation and itch (He and Geha 2010) (Fig. 3).

### Neuropeptide

**Fig. 4:**
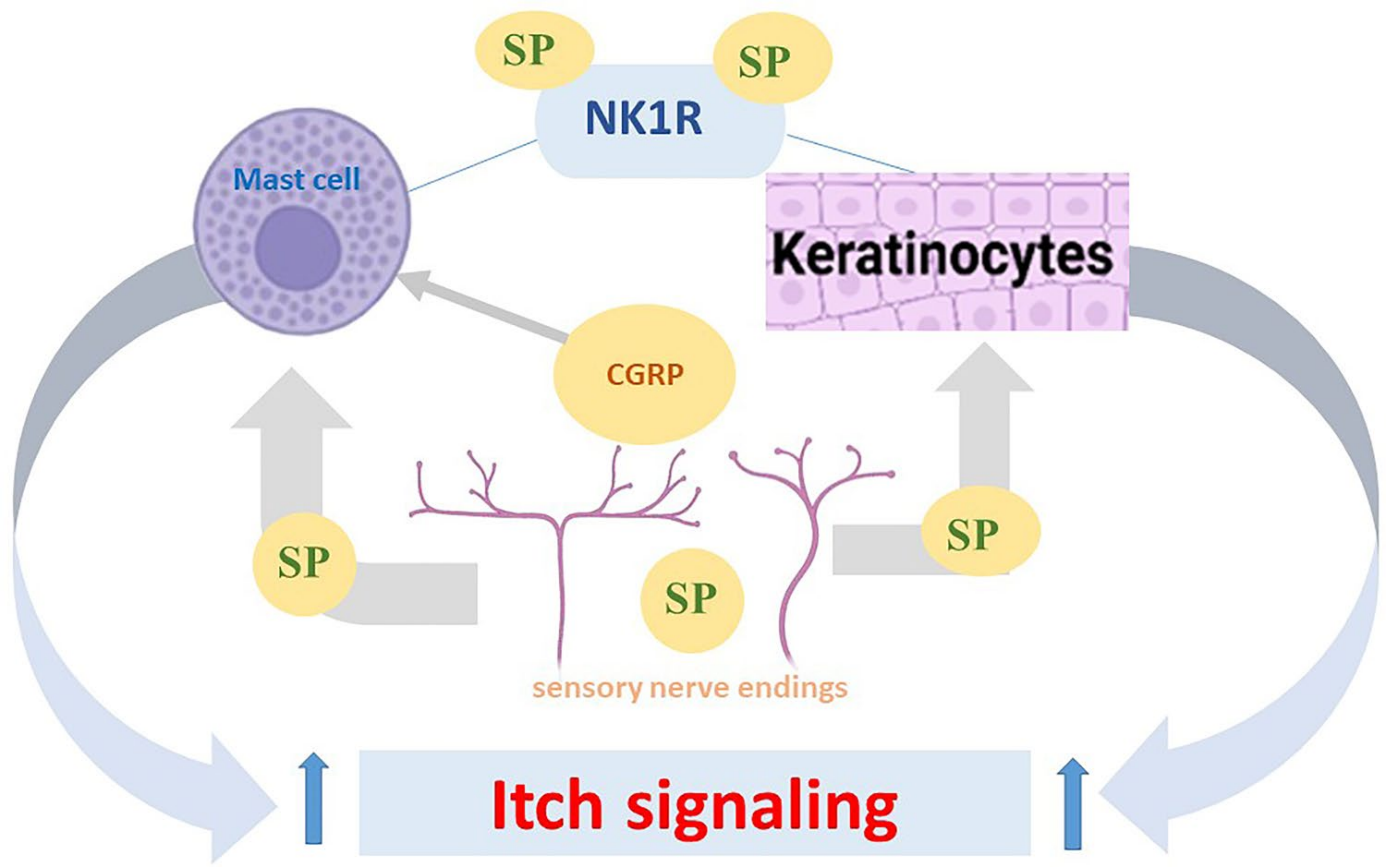
Neuropeptides triggering itch signals in pruritus

Substance P (SP) and calcitonin gene-related peptide (CGRP) are two neuropeptides released from sensory nerve endings in the skin. There is bidirectional communication between nerve fibers and keratinocytes (Fig. 1). Substance P stimulates keratinocytes through neurokinin receptor 1 (NK1R) (Yosipovitch et al. 2018b) to produce cytokines and exacerbating itch. Neuropeptides can also interact with immune cells, leading to the release of pro-inflammatory cytokines and other mediators that intensify itching. SP can bind to NK1R on mast cells, leading to the release of histamine and other substances that promote itching (Marek-Jozefowicz et al. 2023). SP can activate sensory neurons, increasing their sensitivity and spontaneous activity to trigger itch (Shao et al. 2023). Calcitonin gene-related peptide (CGRP) is the predominant neuropeptide in human skin and frequently coexists with substance P (SP)(Wallengren, 2019). CGRP activates mast cells and other immune cells to produce pruritogen mediator which, amplifying itch signaling (Shao et al. 2023) (Fig. 4).

### Protease and protease-activated receptors (PARs)

Protease-activated receptor 2 (PAR2), expressed on sensory nerve endings, is activated by proteases such as tryptase, trypsin, and kallikreins, as well as exogenous proteases. This activation induces the release of neuropeptides, from sensory neurons, which subsequently transmit itch signals to the central nervous system (Paus et al. 2006). PAR2 is also expressed on keratinocytes. When activated, it can disrupt the skin barrier, making it more susceptible to irritants and allergens that cause itching (Buhl et al. 2020). Tryptase, released from mast cells during allergic reactions, activates PAR2 on sensory nerve endings, contributing to itch (Paus et al. 2006) (Fig. 2).

### Central pathways involved in itch sensation

#### Itch signaling in spinal cord

**Fig. 5:**
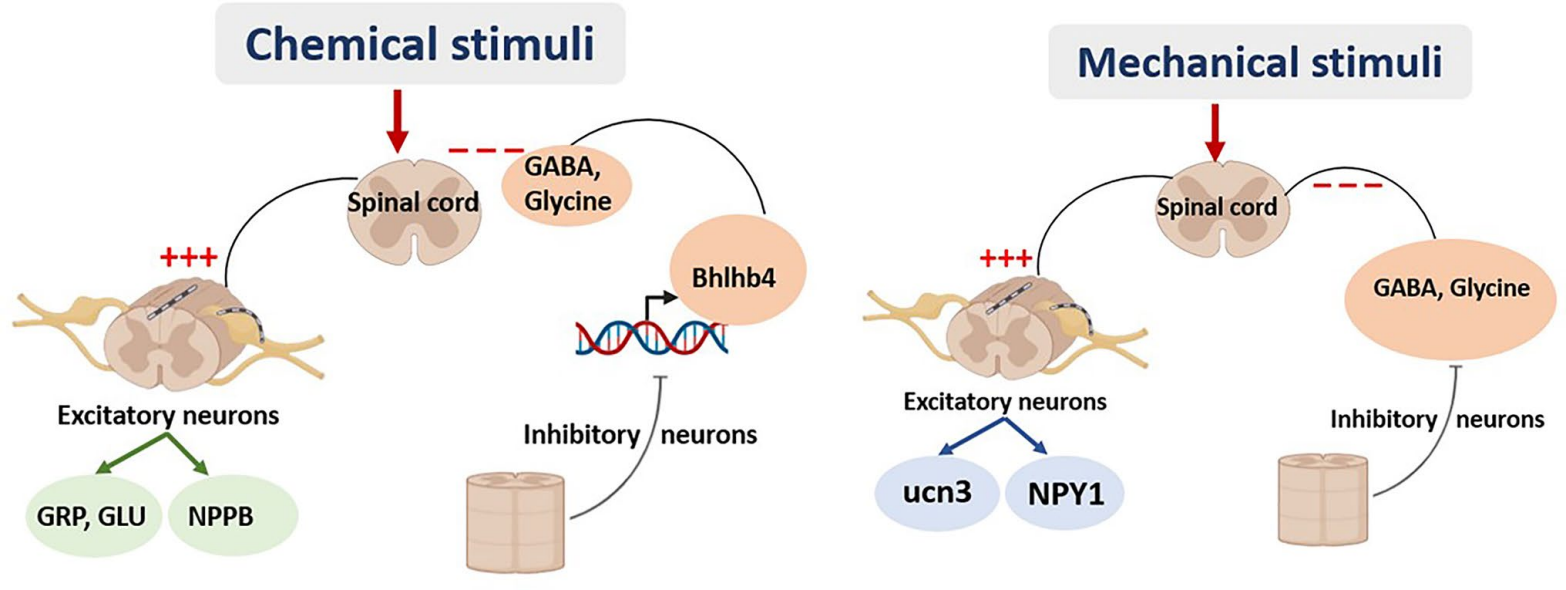
Chemical and mechanical stimuli—spinal cord itch signaling

Itch sensation is initially transmitted from the pruriceptors to the spinal cord via dorsal root ganglia, synapsing with second-order projections in the dorsal horn of the spinal cord (Mahmoud et al. 2023). Itch signaling in the spinal cord involves a complex interplay of excitatory and inhibitory mechanisms that modulate the perception of itch. These signals are modulated by interneurons in the dorsal horn of the spinal cord, which can either amplify or inhibit the itch sensation. Chemical and mechanical itch are processed through distinct spinal pathways. For chemical itch, excitatory neurons expressing the gastrin-releasing peptide receptor (GRPR) and natriuretic polypeptide b receptor (Npra) are mainly involved in the processing of itch (Chen and Sun 2020). When the pruriceptors are activated by chemical stimuli, they send a signal to the spinal neuron by releasing natriuretic polypeptide b (NPPB), which act on Npra. Nppb/Npra signaling forms an upstream pathway activating GRPR neurons by releasing GRP and Glu (Chen and Sun 2020). The chemical itch circuit is controlled by inhibitory interneurons express the transcription factor Bhlhb5, which release GABA, glycine and dynorphin to regulate excitatory neurons and inhibit GRPR + or downstream neurons of the itch pathway (Dong and Dong 2018). Mechanical itch, on the other hand, involves excitatory neurons expressing urocortin 3 (Ucn3) and neuropeptide Y1 receptor (NPY1R), which receive input from low-threshold mechanoreceptors (LTMRs). The Mechanical itch is controlled by inhibitory interneurons expressing neuropeptide Y (Pan et al. 2019) through NPY-NPY1R signaling as well as the inhibitory neurotransmitters GABA and glycine (Chen and Sun 2020) (Fig. 5).

#### Itch signaling in the brain

Itch signals are transmitted from the spinal cord to the brain through spinothalamic and spinoparabrachial tracts (Vander Does et al. 2023). These spinal projection neurons extend their axons to both the thalamus, which serves as a critical relay station for sensory information, and the parabrachial nucleus (PBN) in the brainstem, which plays a significant role in the modulation and integration of these sensory signals (Dong and Dong 2018). Several brain regions are activated by pruritic stimuli, itch-associated scratching, or emotional changes. Despite some variations across studies, common areas of activation include the thalamus, primary and secondary somatosensory cortex (S1 and S2), prefrontal cortex (PFC), anterior cingulate cortex (ACC), insular cortex, premotor and motor cortex, and parietal cortex. Each of these regions is thought to contribute to different aspects of itch processing. The somatosensory cortex encodes the spatial, temporal, and intensity aspects of itch. Motor areas are involved in planning and executing itch-induced scratching behavior. Higher-order cortices, including the PFC and ACC, process the emotional and motivational components of itch. In rodent studies, itch-selective spinal GRP + neurons have been identified in several brain areas, such as the thalamus, PBN, amygdala, S1, periaqueductal gray (PAG), and rostral ventromedial medulla (RVM), as being involved in itch processing (Yosipovitch et al. 2018b; Chen and Sun 2020; Mahmoud et al. 2023). The periaqueductal gray (PAG) region of the brain contains a specific group of neurons that express the gene tachykinin 1 (Tac1). These Tac1-expressing neurons play a crucial role in modulating itch through a descending pathway that extends from the brain to the spinal cord (Gao et al. 2019). Noradrenaline and serotonin (5-HT) play crucial roles as descending neurotransmitters in the modulation of itch and other sensory signals within the central nervous system. Noradrenaline acts on α1-adrenoceptors located on inhibitory interneurons and α2-adrenoceptors on the central terminals of primary sensory neurons in the spinal cord. This dual action enhances inhibitory control over itch transmission, thereby reducing the sensation of itch and the urge to scratch (Kuraishi 2015), serotonin primarily acts through 5-HT1A receptors, which are co-expressed with GRPR in spinal cord neurons. Activation of these receptors can enhance the excitability of GRPR-expressing neurons, thereby modulating itch signals (Zhao et al. 2014).

## Types of itch responses

**Fig. 6:**
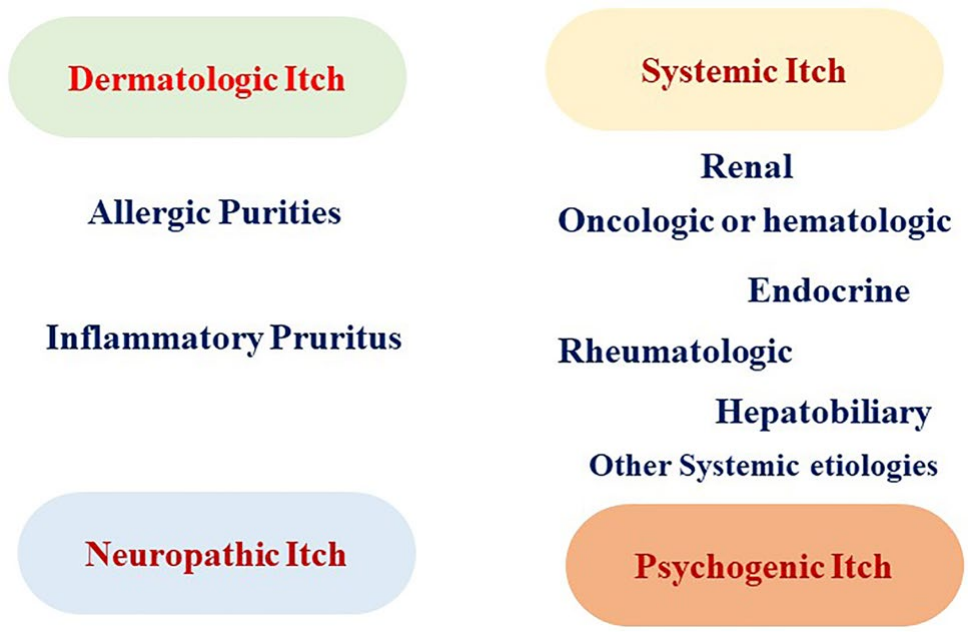
Types of itch responses

Itch is not exclusively linked to allergic or immune-related conditions. According to the well-known study; “International Forum for the Study of Itch”, it is categorized into four clinical types, independent of underlying pathophysiologic mechanisms (Stander et al. 2007): (1) dermatologic itch, resulting from skin disorders such as allergies, inflammation, infections, or insect bites; (2) systemic itch, associated with non-skin conditions like liver, kidney, blood disorders, or medication side effects; (3) neuropathic itch, caused by neurological conditions (Yosipovitch and Samuel 2008; Ständer and Schmelz 2020); and (4) psychogenic itch, linked to psychiatric disorders (Yosipovitch and Samuel 2008) (Fig. 6). Additionally, itch of mixed origin is commonly observed.

## Dermatologic itch

### Allergic purities

Urticaria, also known as hives, is driven by the direct action of histamine, a well-established trigger for pruritus, on sensory nerve fibers (Klein and Clark 1999). This is mediated by mast cells, which are the primary storage sites for various bioactive and immunomodulatory substances in the skin, such as chemokines, cytokines, histamine, serotonin, and tryptase (Metcalfe et al. 1997; Wernersson and Pejler 2014). Upon activation, they release these compounds, influencing processes like tissue remodeling, immune responses, vascular regulation, and nociception (Bischoff 2007; Abraham and St. John 2010). In the context of itch driven by nociceptive mechanisms, mast cells play a critical role by releasing histamine, which, in turn, activates receptors on sensory neurons in the dorsal root ganglia responsible for itch perception (Shim and Oh 2008). Traditionally, mast cell activation occurs when an antigen binds to immunoglobulin E (IgE) antibodies (Galli and Tsai 2012), leading to crosslinking of the high-affinity Fc epsilon RI (FcεRI), IgE receptor, with IgE antibody.

Allergic contact dermatitis (ACD)-related pruritus (Adler and DeLeo 2021), on the other hand, is often triggered through biological pathways that do not rely on histamine release from mast cells. Notably, through Mas-related G-protein-coupled receptors (Mrgprs) such as Mrgpr2. Mrgprb2 activation, in contrast to FcεRI, leads to the release of higher levels of tryptase and lower amounts of monoamines such as histamine and serotonin. Additionally, Mrgprb2 activation in mast cells specifically stimulates non-histaminergic sensory neurons, which is a distinct population of sensory neurons that do not depend on histamine receptors (Meixiong et al. 2019a).

### Inflammatory pruritus

Cellulitis and pityriasis lichenoides, inflammatory skin conditions, could or could not result in itching. Meanwhile, psoriasis and atopic dermatitis (AD), frequently lead to moderate-to-severe itching in the majority of patients. Given that AD is both common and consistently associated with itch (Wong et al. 2017). AD is considered to be the prototypic inflammatory skin disorder that is invariably linked to intense itching (Buddenkotte and Steinhoff 2010). It is driven by cytokines such as IL-4, IL-13, and IL-31, which can function as pruritogens—substances that trigger itch when applied to the skin—and activate Janus kinase (JAK) 1 signaling in itch-sensitive nerves (Oetjen et al. 2017; Campion et al. 2019; Miron et al. 2022; Mack et al. 2023).

Infectious causes of itch, such as tinea corporis or ectoparasitic infestations like scabies, result in inflammatory itching. In these cases, the infectious organism triggers pruritus by provoking an immune response from the host.

## Systemic itch

### Renal

Renal itch commonly occurs in late stages of chronic kidney disease (CKD), affecting between 40 to 90% of hemodialysis patients (Combs et al. 2015; Hu et al. 2018). It is linked to systemic inflammation, uremic neuropathy, and imbalances in opioid receptor activity (Kumagai et al. 2010). Secondary hyperparathyroidism may also contribute, as itch has improved in some patients after parathyroidectomy (Massry et al. 1968; Chou et al. 2000).

### Hepatobiliary

Cholestasis from hepatobiliary conditions is a common cause of itch. It results from bile acid buildup due to primary or secondary biliary obstruction, such as intrahepatic cholestasis of pregnancy, primary biliary cholangitis, sclerosing cholangitis, viral hepatitis, and cirrhosis (Weisshaar and Dalgard 2009; Bunchorntavakul and Reddy 2012; Patel et al. 2019). Cholestatic itch involves lysophosphatidic acid, bile acids, bilirubin, and altered opioid receptor activity (Meixiong et al. 2019b; Patel et al. 2019). Bilirubin may trigger pruritus through Mas-related G-protein-coupled receptor (Mrgprx4) on sensory neurons (Bunchorntavakul and Reddy 2012). It often starts on the soles and palms. And it becomes widespread as the disease progresses.

### Endocrine

Pruritus is relatively more common in diabetic patients than in healthy individuals (26.3% vs. 14.6%). Diabetes increases the risk of itch-related conditions like fungal infections, excoriation disorder, neuropathy, and scalp or vulvar pruritus (Stawiski and Voorhees 1976; Scribner 1977; Neilly et al. 1986; Kwon et al. 2020). Itch in diabetes may result from nerve damage caused by high glucose levels, linked to diabetic polyneuropathy. Uncontrolled hyperthyroidism can also cause itches in some patients due to a lower itch threshold from increased temperature, vasodilation, and kinin activation (Caravati et al. 1969; Krajnik and Zylicz 2001). Hypothyroidism is less often associated with itch but is linked to xerosis (Krajnik and Zylicz 2001).

### Rheumatologic

Itch is a frequent symptom in rheumatologic diseases, driven by immune system activation (Yahya 2019). About half of systemic sclerosis patients experience pruritus, often alongside xerosis (Razykov et al. 2013). In dermatomyositis, 50.8% report moderate-to-severe itching (Kim et al. 2018), which correlates with skin involvement (Robinson et al. 2015). Other autoimmune conditions associated with varying levels of itch include Sjögren syndrome, cutaneous and systemic lupus erythematosus (Valdes-Rodriguez et al. 2017).

### Oncologic or hematologic

Itch is frequently observed in hematologic malignancies (Weisshaar and Dalgard 2009). It affects up to 30% of patients with Hodgkin lymphoma (Gobbi et al. 1983), 15% with non-Hodgkin lymphoma (Mandal et al. 2007), and 67% with polycythemia vera. Polycythemia vera patients often experience aquagenic pruritus (Lelonek et al. 2018), triggered by water contact at all temperatures. Generalized itch, sometimes accompanied by eczematous, urticaria, or lichenified skin changes, can occur in conditions like hypereosinophilic syndrome (Leiferman et al. 2007), marked by elevated eosinophil counts (Valent et al. 2012). Pruritus is also linked to cutaneous lymphomas, dermatologic cancers, and hepatobiliary malignancies. Although rare cases of itch have been reported in solid tumors such as insulinoma, non-small cell lung carcinoma, and gastric carcinoid tumors (Larson et al. 2019).

### Other systemic etiologies

Itch can be a side effect of medications like immune checkpoint inhibitors (Le et al. 2022), opioids (Lipman and Yosipovitch 2021), and antimalarials (Ajayi et al. 1989). It may also result from iron-deficiency anemia (Takkunen 1976), heavy metal exposure (Patel et al. 2020), vitamin deficiencies (Umar et al. 2018), HIV (Kaushik et al. 2014), or other viral infections. Low vitamin D and B12 levels are linked to pruritic conditions like dermatitis and psoriasis (Tuchinda et al. 2018), with limited benefit from supplements. In HIV, 13–45% of patients experience chronic itches, often alongside conditions like seborrheic dermatitis and eosinophilic folliculitis (Rodwell and Berger 2000; Dlova and Mosam 2006; Huang et al. 2020).

## Neuropathic itch

Neuropathic pruritus (NP) is a challenging and under-researched type of chronic itch. It is defined as an itch resulting from damage to the somatosensory nervous system. NP represents about 8% to 19% of chronic pruritic skin conditions and encompasses a broad range of neurological disorders (Stander et al. 2007; Yosipovitch and Samuel 2008).

Brachioradial pruritus typically affects middle-aged women with lighter skin, and it worsens more with sun exposure (Pinto et al. 2016; Steinhoff et al. 2018). It begins with itching or burning along the upper extremities and shoulders (C3–C7 dermatomes) and is linked to cervical spine degeneration (Marziniak et al. 2011; Mirzoyev and Davis 2013; Weinberg et al. 2018).

Notalgia paresthetica causes a unilateral itch near the scapula, originating from T2–T6 nerve entrapment. It correlates with spinal changes and reduces nerve density from chronic scratching. Scalp dysesthesia involves discomfort in the scalp, linked to degeneration at C2–C7 levels. Anogenital pruritus is associated with lower spine degeneration at L4–S2 (Savk and Savk 2005; Huesmann et al. 2012).

Other conditions causing neuropathic itch include trigeminal trophic syndrome, strokes, brain infections, and small fiber neuropathies (Steinhoff et al. 2018).

In NP, nerve fiber degeneration may increase proinflammatory mediators like cathepsin S, lysophosphatidic acid, IL-31, and IL-33, which activate itch-specific neurons. Sensory neurons release neuropeptides such as SP, CGRP, glutamate, and nerve growth factor, promoting neurogenic inflammation. Sensitizing TRPV1 to SP and CGRP, through nerve growth factor binding to tropomyosin receptor kinase A (Pereira et al. 2022; Sutaria et al. 2022).

Proinflammatory mediators in inflammatory conditions can influence NP as well. For instance, IL-4 levels rise in nerve stumps after sciatic nerve injury. Nerve damage may increase excitability by upregulating voltage-gated sodium channels, TRPV1 receptors, and TRPM8 receptors. Moreover, gain-of-function mutations in sodium channels like NA_V_1.7 and NA_V_1.9 are linked to severe NP (Pereira et al. 2022).

## Psychogenic itch

Psychogenic itch is characterized as a condition where itching is the primary symptom, with psychological factors significantly influencing its onset, severity, worsening, or persistence. This condition is not well recognized by either psychiatrists or dermatologists. The exact prevalence remains unclear due to challenges in establishing a differential diagnosis (Szepietowski and Reszke 2016).

Itch is frequently experienced by individuals with anxiety and depression, though its underlying mechanisms remain unclear. The itch severity is often linked to and associated with the intensity of the depressive symptoms (Gupta et al. 1994). Chronic pruritus significantly impacts sleep and quality of life (Kaaz et al. 2019; Patel et al. 2021), contributing to greater psychiatric challenges and a higher likelihood of suicidal thoughts (Dalgard et al. 2020). It is also commonly observed in primary psychodermatologic conditions, such as dermatitis artefacta, obsessive–compulsive disorder, delusional infestation, somatic symptom disorder, Morgellons disease, and excoriation disorder. Excoriation disorders have associations with type 2 diabetes mellitus, anxiety disorders, as well as depression (Kwon et al. 2020). Additionally, chronic itch can result from substance use disorders involving opioids, cocaine, or methylenedioxymethamphetamine (MDMA) (Schneider et al. 2006).

### Mechanisms of action of natural antipruritic agents

Natural antipruritic agents exert their effects through diverse and interconnected mechanisms that target the underlying causes of itch at multiple levels. Numerous Studies have shown that bioactive compounds derived from natural sources influence key itch pathways by modulating immune mediators, inhibiting inflammatory cascades, and directly affecting sensory neurons responsible for transmitting itch signals (Vaia et al. 2016; Guo et al. 2018; Kim et al. 2022). By suppressing cytokine release and blocking cyclooxygenase pathways, these compounds reduce the inflammatory environment that exacerbates chronic pruritus (Wu et al. 2021; Elkhawaga et al. 2023). At the same time, many natural agents stabilize mast cells or act as histamine receptor antagonists, effectively mitigating histamine-driven itch (Oliveira et al. 2004; Yang et al. 2021; Saini and Dhiman 2022). Beyond these immune-mediated actions, some bioactive compounds target neural circuits, modulating transient receptor potential channels and other ion channels involved in sensory signaling (Calixto et al. 2005; Stotz et al. 2008). Together, these mechanisms allow natural antipruritic agents to offer a multidimensional approach to itch relief. This integrated mode of action makes them particularly promising for conditions resistant to conventional therapies, as they some of these compounds combine anti-inflammatory effects, antihistaminic properties, and neural modulation to alleviate itch. Here, in this section of our review, we discuss three principal mechanisms by which these agents alleviate itch: anti-inflammatory properties, antihistaminic effects, and modulation of pain pathways.

### Anti-inflammatory properties of natural antipruritic agents

An inflammatory cycle damages the skin barrier, perpetuating a loop of pruritus and inflammation. Some natural antipruritic agents break this cycle by modulating inflammatory pathways, stabilizing immune responses, and restoring skin homeostasis (Subedi et al. 2020). Curcumin, the active compound of *Curcuma longa* (turmeric), is a well-documented natural anti-inflammatory agent that targets multiple inflammatory pathways. Curcumin inhibits NF-κB signaling, a key pathway regulating cytokines like IL-1β, TNF-α, and IL-31, thereby reducing inflammation at the molecular level (Jobin et al. 1999; Buhrmann et al. 2011; Lee et al. 2012). Additionally, it mitigates oxidative stress by scavenging reactive oxygen species (ROS), preventing oxidative damage that exacerbates chronic inflammation. In conditions like atopic dermatitis and psoriasis, curcumin has been shown to reduce redness, scaling, and itching, leading to significant in clinical and preclinical studies (Antiga et al. 2015; Sharma and Naura 2020; Saini et al. 2022). Another effective natural agent is Aloe vera, known for its rich composition of polysaccharides, glycoproteins, and vitamins, which contribute to its anti-inflammatory activity. Aloe vera suppresses prostaglandin E2 (PGE2) synthesis by inhibiting the cyclooxygenase (COX) pathway, thereby reducing swelling, erythema, and pruritus (Agung et al. 2016). Additionally, it stabilizes mast cells to prevent histamine release, a key mediator of inflammation and itch (Budai et al. 2013). In vitro studies have shown that aloe vera gel reduces cytokine levels such as IL-6 and TNF-α, while clinical trials support its effectiveness in alleviating itch and inflammation in conditions like seborrheic dermatitis and sunburn-induced pruritus (Habeeb et al. 2007; Wang et al. 2023). Another example is Chamomile extract (*Matricaria chamomilla*) further complements these anti-inflammatory effects with its bioactive components, such as apigenin, bisabolol, and chamazulene (Ortiz et al. 2016; El Mihyaoui et al. 2022). Apigenin suppresses NF-κB activation and reduces the production of pro-inflammatory cytokines. Chamomile also supports the repair of the skin barrier by promoting keratinocyte proliferation and differentiation, which indirectly reduces pruritus caused by barrier dysfunction (Lin et al. 2018). Clinical studies have demonstrated the effectiveness of chamomile in managing eczema, significantly improving itch severity and skin hydration (Weber et al. 2014; Weir et al. 2016).

### Antihistaminic effects of natural antipruritic agents

Despite synthetic antihistamines targeting H1 and H4 receptors are widely used, their side effects, such as sedation and dizziness limit their long-term application (Kollmeier et al. 2014). Natural antihistaminic agents, however, offer a safer alternative with the added benefit of immune modulation, making them effective in alleviating histamine-induced pruritus. Among the most studied natural antihistaminic agents is quercetin, a flavonoid found abundantly in onions, apples, and green tea (López-Enríquez et al. 2023). For example, quercetin effectively inhibits histamine’s actions by antagonizing H1 receptors, preventing sensory neurons from being depolarized and blocking itch transmission (Weng et al. 2012). Moreover, quercetin exerts a stabilizing effect on mast cells, inhibiting degranulation and significantly reducing the release of histamine, tryptase, and prostaglandins, all of which are central to the histamine-driven inflammatory cascade. Beyond receptor antagonism, quercetin also suppresses histidine decarboxylase (HDC), the enzyme responsible for histamine synthesis, reducing overall histamine levels in inflamed tissues and mitigating chronic pruritus (Castellani et al. 2007). By targeting multiple points in the histamine pathway, quercetin not only reduces itching but also addresses the inflammatory milieu that exacerbates pruritic conditions. In addition to quercetin, stinging nettle (*Urtica dioica*), a traditional remedy long used for allergic symptoms, demonstrates remarkable antihistaminic properties. Rich in bioactive compounds like chlorogenic acid and flavonoids, stinging nettle works by competitively binding to H1 receptors, thereby blocking histamine activity and alleviating histamine-induced itch and redness (Roschek et al. 2009). Furthermore, nettle stabilizes mast cells, preventing the release of histamine and curtailing the downstream inflammatory effects (Grauso et al. 2020). Its antihistaminic effects are further complemented by its broader anti-inflammatory activity, including inhibition of COX-1 and COX-2 enzymes, which play critical roles in inflammatory responses. Clinical studies have validated the efficacy of stinging nettle, demonstrating significant relief of symptoms in conditions like allergic rhinitis compared to placebo (Bakhshaee et al. 2017). These findings highlight stinging nettle as a promising natural option for managing histamine-driven pruritus while offering additional anti-inflammatory benefits. By targeting histamine receptors, stabilizing mast cells, and reducing histamine synthesis, natural agents such as quercetin and stinging nettle provide a multidimensional approach to combating histamine-induced itch. Their ability to alleviate pruritus without the sedative effects associated with synthetic antihistamines underscores their therapeutic potential as safe and effective alternatives in managing allergy and inflammatory skin conditions.

### Modulation of pain pathways by natural antipruritic agents

Itch and pain share significant overlaps within the somatosensory system, both being transmitted by specialized nerve fibers and processed through common pathways in the spinal cord and brain (He et al. 2016; Follansbee et al. 2021). Despite their distinct perceptions, these sensations interact through complex mechanisms. Itch signaling primarily involves unmyelinated C-fibers, which respond to pruritogens via specific receptors like TRPV1 (transient receptor potential vanilloid 1), TRPA1 (transient receptor potential ankyrin 1), and histamine-sensitive pathways (Kim et al. 2004; Wang et al. 2011; Wilzopolski et al. 2021). Interestingly, the activation of nociceptive pain fibers, such as Aδ fibers and C-fibers, can suppress itch signals through inhibitory interneurons in the spinal cord. This phenomenon explains why scratching an action that activates pain pathways can temporarily relieve itch (Mochizuki and Kakigi 2015; Schmelz 2015; Snyder and Ross 2015; Dong and Dong 2018). By targeting shared neural pathways, natural agents offer an effective strategy to reduce pruritus without triggering significant pain (Paus et al. 2006; Fernández-Carvajal et al. 2022). One such agent is capsaicin, the bioactive compound in chili peppers, which specifically targets the TRPV1 receptors expressed on C-fibers. Initially, capsaicin activates TRPV1, inducing a transient burning or stinging sensation (Knotkova et al. 2008). However, with prolonged exposure, the receptors become desensitized, reducing the excitability of itch-transmitting nerve fibers and dampening pruritic signaling (Knotkova et al. 2008; Sekine et al. 2012; Melo et al. 2018). This desensitization mechanism makes capsaicin particularly effective for neuropathic pruritus, psoriasis-associated itch, and chronic itch syndromes where conventional antihistamines often fail. Clinical studies have demonstrated the significant efficacy of topical capsaicin formulations in reducing itch severity and improving patient outcomes, especially in some cases like hemodialysis-related pruritus (Ellis et al. 1993; Tarng et al. 1996; Andersen et al. 2017; Thangam et al. 2018). Another natural compound, menthol, derived from peppermint oil, exerts its antipruritic effects by activating TRPM8 receptors, which are cold-sensitive channels on sensory neurons (Bautista et al. 2007). TRPM8 activation produces a distinct cooling sensation that competes with itch signals at the central nervous system level, effectively reducing itch perception (Palkar et al. 2018; Yin et al. 2022) (Table 2).

**Table 2:** Key natural sources as antipruritic agents

	Plant name	Type of extract/bioactive compound	Part used	Type of study		Results	Refs
Plants (a- herbal extracts)							
	*Rheum rhabarbarum*, *Kniphofia foliosa, Aloe barbadensis*, and *Polygonum multiflorum Thunb*	Aloe-emodin (anthraquinone derivative)		**In vivo** • Neck model of acute pruritus • allergic contact dermatitis model		Aloe-emodin inhibited mast cell degranulation in skin lesions and suppressed the mRNA expression of inflammatory cytokines, such as IL-4, and IL-6	(Yang et al. 2024)
	*Aloe barbadensis, arborescens, forex, Africana, saponeria* and aloe powder of Japanese pharmacopia	Aqueous extract and barbaloin (isolate)	Leaves	***In-vivo*** Antigen and compounds 48/80 induced histamine release from rat peritoneal mast cells		• Aloes extracts and barbaloin inhibited release of histamine induced by IgE antibody and compounds 48/80 and chemical mediators from mast cells • Barbaloin inhibited mast cell degranulation via inhibition of influx of extracellular Ca^2+^ and its mobilization	(Yamamoto et al. 1993)
		Acemannan (polysaccharide), flavonoid (phenolic), bradykinase (enzyme), salicylic acid	Unorganized drug (Gel)	*Clinical trial*		• Aloe vera’s healing power mainly comes from its polysaccharide acemannan, which boosts skin cell activity and collagen production, speeding up wound healing and calming inflammation. It also has glycoproteins and growth factors that reduce inflammation by blocking key inflammatory molecules like IL-6 and TNF-α. Plus, its salicylic acid offers mild pain relief and anti-itch effects. The gel’s water content hydrates skin, while its polysaccharides create a protective, moisture-locking barrier against irritants	(Hekmatpou et al. 2019)
	*Matricaria recutita, M. chamomilla *(chamomile)	Ethyl acetate extract and essential oil nanoemul gel	Flowers	***In-vivo*** • Compound 48/80-induced scratching • Capsaicin induced atopic dermatitis in rats		• Inhibited scratches • Co-administration with antihistamine H1 antagonists (oxatomide and fexofenadine exhibited a significant synergistic antihistaminic effect • Aid in treatment of eczema and dermatitis • Topical nanoemul gel application IL-4, IL-18 and IL-22. Improved skin, reduced oedema and inflammatory cells	(Kobayashi et al. 2005; El-Salamouni et al. 2020; Akram et al. 2024)
	*Calendula officinalis* (calendula)	Hydro-alcoholic extract formulated to Topical cream (W/O emulsion)	Peeled plant	**Single clinical blinded trial** Assessment of skin condition		• ↓Skin erythema • ↓Trans-epidermal water loss	(Akhtar et al. 2011)
		fatty acids, saponins, flavonoids, and triterpenoid compounds	Flower head	*Clinical trials*		Flower head oil is distinguished by its high content of fatty acids, which contribute to the restoration and reinforcement of the skin’s barrier function and provide photoprotective effects against ultraviolet radiation. Additionally, the saponins, flavonoids, and triterpenoid compounds present in flowers exhibit potent anti-inflammatory properties, mitigating inflammatory responses and alleviating clinical manifestations such as erythema, pruritus, and edema commonly observed in conditions like psoriasis and dermatitis. Flower’s natural anti-inflammatory and skin-healing properties make it a good choice to relieve itch Applications: a. Vaginal gels with calendula eased itching, dryness, and irritation in vaginal dystrophy b. Creams helped heal diabetic foot infections and itching • Oil mixtures containing calendula improved dry, itchy skin in the elderly better than coconut oil	(Tedeschi et al. 2012; Cioinac 2016; Yahya et al. 2021)
	*Curcuma longa Curcuma aromatic* (turmeric)	• Turmeric capsules Curcuminoids (curcumin, demethoxycurcumin and bisdemethoxycurcumin)	Rhizomes	• **Double-blind randomized clinical trial** treating uremic pruritus in hemodialysis patients ***In-vivo*** IgE-antigen complex induced passive cutaneous anaphylaxis (PCA) and compound 48/80 induced scratching		• ↓ pruritus score • ↓ hs-CRP level • curcuminoids inhibited the PCA reaction induced by IAC and scratching behavior in mice • Curcuminoids repressed degranulation, protein expression of TNF-α and IL-4, and activation of NF-κB in IgE-antigen complex induced RBL-2H3 cells • Improved symptoms of itching and anaphylaxis	(Trinh et al. 2009; Lee et al. 2018; Kumar et al. 2023)
	*Glycyrrhiza glabra* (licorice)	Glycyrrhizic acid, Licochalcone A, glabridin and liquiritin	Root and rhizome	***In-vivo*** • Relieving atopic dermatitis • IgE-mediated passive and systemic anaphylaxis in mice		• ↓IgE in serum, ear swelling, reduced the infiltration of mast cells in skin lesions • ↓ Expressions of IL-4, IFN-γ, TNF-α and thymic stromal lymphopoietin in skin lesions • Th1/Th2/Th17-immune responses in the draining lymph node • ↓ TNF-α and MCP-1	(Hou et al. 2022; Shu et al. 2022)
				*Clinical trials*		Glycyrrhizin inhibits COX and phospholipase A2 enzymes → reduces prostaglandins and leukotrienes → lowers inflammation; downregulates TNF-α and IL-6; inhibits histamine release from mast cells; antioxidant flavonoids (glabridin, liquiritin) reduce ROS and oxidative stress, protecting skin cells. relief from itchiness, especially in conditions like eczema and dermatitis also treating itch associated with allergies • Applied as Licorice extract topical cream (1–2%)	(Hoffmann et al. 2020; Hasan et al. 2021)
	*Forsythia suspensa*	Forsythoside B	Flowering shrub	*In-vivo*		• selectively inhibits the TRPV3 channel, putatively by blocking ion passage. Potential treatment for alleviating pruritus	(Kim et al. 2021)
	*Avena sativa* (Oat)	Avenanthramides (phenolic compounds)	Seed	*In-vitro*		• Inhibited the degradation of inhibitor of nuclear factor kappa B-α (IκB-α) in keratinocytes with subsequent reduction of interleukin-8 (IL-8) release	(Sur et al. 2008; Cerio et al. 2010)
				*In vivo* (murine model)		• Contact hypersensitivity and neurogenic inflammation and reduced pruritogen-induced scratching in a murine itch model	
	*Oenothera biennis*	Gamma-linolenic acid (GLA), linoleic acid, phenolic compounds, flavonoids	Seeds (seed oil)	*Clinical trials*		• GLA converts to DGLA, producing anti-inflammatory prostaglandin E1 (PGE1), reducing skin inflammation by inhibiting leukotrienes and interleukin pathways; linoleic acid maintains skin barrier and hydration; antioxidants reduce oxidative stress, improving pruritus and skin health. Clinical studies show mixed but promising antipruritic effects, especially in eczema and atopic dermatitis	(Chung et al. 2018; Timoszuk et al. 2018; Sharifi et al. 2024)
	*Lactuca sativa* L (lettuce)	γ-tocopherol, δ-tocopherol, campesterol, α-tocopherol, α-lactucerol	Leaves and seeds	*In-silico*		• Showed the highest binding affinity to human kappa opioid receptor (DS = -11.72 kcal/mol, Ki = 2.56 nM), acting as a kappa opioid receptor antagonist, with anti-inflammatory, antipruritic, and analgesic predicted activities. Demonstrated hydrophobic and hydrogen bond interactions with key residues on receptor; superior to gabapentin, suggesting effective antipruritic potential through opioid receptor modulation. Also, antioxidants and anti-inflammatory properties aid symptom relief in uremic pruritus	(Sepehri et al. 2022)
	*Perilla frutescens* L. (Perilla)	Luteolin (flavonoid)	Leaves	*In-vivo*		• Luteolin reduced histamine release and scratching by blocking inflammatory cytokines from mast cells	(Jeon et al. 2014)
	*Pistacia lentiscus*	Mastic	Unorganized drug	*In-vivo*		• Mastic reduces itch behavior due to pro-inflammatory cytokine production in dose-dependent manner	(Kishimoto et al. 2021)
	Curly kale extract	30% kaempferol flavonoids	–	*Clinical trial*		• Extract with very high antioxidant capacity reduces expression of inflammatory cytokines in keratinocytes, so they reduce itching,	(Zhang et al. 2022)
	Green tea extract	60% epigallocatechin gallate					
	Apple extract	15% phlorizin, 5% quercetin flavonoids					
Plants (b- essential oils)							
	*Lavandula Officinalis* (lavender)	Essential oil Topical cream	Flowers	**Randomized clinical trial** Reducing the eczema severity		• Marked ↓ in severity of eczema symptoms compared to hydrocortisone • ↓ Itching and redness in pregnant women due to scratched wounds • Linalool inhibited the degranulation of mast cells and thickening of the epidermal layer	(Rashidipour et al. 2023) (Asih et al. 2021)
	*Lavandula angustifolia*	Essential oil; linalool, linalyl acetate, 1,8-cineole, β-caryophyllene	Flower head	*In-vivo*		• Exhibits anti-inflammatory, analgesic, antioxidant, and antiseptic effects by inhibiting pro-inflammatory cytokines (IL-6, TNF-α), modulating opioid and serotonergic receptors, stabilizing mast cells, and improving skin barrier function. Demonstrated efficacy in reducing pruritus in hemodialysis, gestational pruritus, atopic dermatitis, and allergic reactions, while promoting wound healing, pain relief in episiotomy and inflammatory skin conditions, and regulating hormonal levels in PCOS patients. Shows comparable efficacy to standard treatments with a favorable safety profile	(Batiha et al. 2023; Kandilarov et al. 2023)
				*Clinical trials*			
	*Syzygium aromaticum* (Clove)	Eugenol (monoterpene)	Flower	*In-vivo*		Eugenol inhibits pro-inflammatory mediators, including IL-1β, IL-6, TNF-α, PGE2, iNOS, COX-2, NF-κB, and 5-LOX, thereby reducing swelling, erythema, and pruritus • Application: eugenol-loaded transethosomal Gel for treatment of Atopic Dermatitis	(Ulanowska and Olas 2021; Kashyap et al. 2024)
	*Matricaria recutita L.* (German Chamomile)	Volatile oil (bisabolol)	Flower head	*In-silico* *In-vivo*		Volatile oil regulated T-cell lymphatic subpopulations to inhibit the Th17 cell differentiation signaling pathway. This resulted in a reduction of (IL-17), thereby inhibiting the activation of the (NF-κB) and MAPK pathways, decreasing the secretion of the pro-inflammatory factors (TNF-α) and (IL-6)) and reducing inflammation	(Wang et al. 2021)
	*Mentha piperita* (peppermint)	Menthol and flavonoids, phenolic acids, triterpenes	Leaves and oil	**Triple-blind clinical trial** Relieving itching in a pregnant woman ***In-vivo*** Suppression psoriasis-induced itching in mice		• Stimulate TRPM8 channel resulting in a cooling effect that opposes itching sensation • ↓ release of histamine and proinflammatory mediators • Safely relieved itching in pregnant women via activation of A-delta fibers and κ-opioid receptors • ↓ IL-10 and TGF-β in mice and ↑ skin elasticity	(Gonçalves et al. 2024) (Amjadi et al. 2012)
	*Melaleuca alternifolia* (Tea tree)	Essential oil	Leaves and terminal branches	**Clinical trial** Treating itching associated with histamine-induced weal and flare		• ↓ Histamine release • Water-soluble components of TTO, especially terpinen-4-ol ↓ TNF-α, IL-1β, 8 and 10, and prostaglandin E_2_	(Koh et al. 2002)
Animal							
	Scientific name/material	Type of study				Results	Refs
	Honey	**Clinical trial** Improving atopic dermatitis				• ↓ IL4 • Suppressed mast cell degranulation	(Alangari et al. 2017)
	Propolis	***In-vitro*** Suppressing atopic dermatitis in HaCaT cells ***In-vivo*** suppressing atopic dermatitis in mice ***Ex-vivo*** Suppressing atopic dermatitis human skin model				• In both HaCaT cells and human skin: - ↓ TNF-α, IL-6 and IL-8 - ↓ Monocyte chemoattractant protein-1 and macrophage-derived chemokine - Improved barrier proteins, filaggrin and involucrin levels • In mice: • ↓ IL-4, IL-13, L-25 and IL-33 • ↓ scratching and transepidermal water loss • Caffeic acid phenethylester is the active compound in propolis	(Cho et al. 2024)
Mineral-based remedies							
	Scientific name/material	Application method			Type of study	Results	Refs
	Colloidal oatmeal	Colloidal oatmeal cream Oatmeal lotion			**Double-blind clinical trial** Alleviating chronic irritant hand eczema **Clinical trial** Prevention of pruritus in hemodialysis patients ***In-vitro*** and ***in-vivo*** Anti-inflammatory and anti-irritant effects of Avenanthramides	• ↓ Hand eczema severity index score and pruritus severity visual analogue scale • Improved Dermatology Life Quality Index (DLQI) • Improved skin dryness, scaling, roughness, and itching • Avenanthramides, phenolic compounds in oats, • Inhibited degradation of inhibitor of nuclear factor kappa B-α in keratinocytes • ↓ TNF-α and IL-8 • Topical applications reduced inflammation, hypersensitivity, neurogenic inflammation and scratching in mice	(Sari 2022) (Sobhan et al. 2020) (Sur et al. 2008)
	Sea salt baths	• Topical Dead Sea salt magnesium ointment • Bathing in 5% Dead Sea salt			• Treating chronic seborrheic and atopic dermatitis • Effect of sea salt bathing in skin inflammation	• Anti-inflammatory, stabilized mast cells and improved symptoms of chronic seborrheic and atopic dermatitis • Bathing for 15 min improved skin barrier function, enhanced stratum corneum hydration, and reduced skin roughness and inflammation	(Proksch et al. 2005) (Sudan 2024a, b)

### Plant-derived antipruritic agents

Pruritus is a painful skin sensation that causes the desire to scratch. Medicinal plants have been recommended as a valuable resource for discovering new bioactive compounds. The purpose of this study was to document certain medicinal plants and their phytochemicals for the treatment of pruritus. Plant-derived molecules have been shown to reduce serum IgE and proinflammatory cytokines, stabilize mast cells, suppress the Th2 cellular response, suppress substance P and NFκB expression, inhibit prostaglandin E2 production, and activate itch receptors. Overall, various medicinal plants and their bioactive constituents have demonstrated significant activity in the management of pruritus and can thus be considered an alternative source of treatment (Mohajerani et al. 2019).

### Bioavailability barriers in botanical antipruritics

The therapeutic potential of natural products for managing pruritus is supported by their complex pharmacology; however, clinical translation is frequently hindered by issues related to bioavailability. Curcumin derived from Curcuma longa demonstrates significant antipruritic effects mainly by inhibiting NF-κB, leading to the downregulation of inflammatory cytokines such as TNF-α and IL-6, and by stabilizing mast cells to inhibit histamine release. Nonetheless, its effectiveness is constrained by very low oral bioavailability resulting from extensive metabolism, which renders topical administration a more feasible option (Prasad et al. 2014; Hewlings and Kalman 2017). Similarly, apigenin and alpha-bisabolol, the primary constituents found in chamomile (*Matricaria chamomilla*), have significant anti-inflammatory and antihistaminic properties when applied topically, but they also increase the risk of contact dermatitis in people who are sensitive to Asteraceae (Srivastava et al. 2010). Because glycyrrhetinic acid inhibits 11β-hydroxysteroid dehydrogenase, licorice (*Glycyrrhiza glabra*) has a corticosteroid-like effect as well as is an effective topical anti-inflammatory. Nevertheless, its oral bioavailability is associated with significant pseudoaldosteronism, which limits its systemic use (Pastorino et al. 2018a). Both calendula (*Calendula officinalis*) and aloe vera (*Aloe barbadensis*) have excellent safety profiles with little irritation, mainly through topical mechanisms. Calendula promotes wound healing and lowers pro-inflammatory cytokines, while aloe vera has a cooling effect and contains anti-kininase enzymes (Reuter et al. 2008; Parente et al. 2012). Peppermint (*Mentha piperita*) and lavender (*Lavandula angustifolia*) essential oils mediate their effects through neuromodulation; menthol activates TRPM8 receptors to produce a cooling sensation that dominates itch signals, while linalool in lavender provides local anesthetic and anxiolytic effects. However, both must be adequately diluted to avoid skin irritation and cytotoxicity (Cavanagh and Wilkinson 2002; Kazemi et al. 2025). Thus, while these botanicals are promising, their application requires careful consideration of delivery methods and potential irritancy to maximize efficacy and safety.

### Adverse effects and risk mitigation of botanical antipruritic agents

Extensive research, including both clinical trials and case reports, has been conducted to evaluate the safety profiles of these natural antipruritics. Although chamomile (*Matricaria chamomilla*) is generally harmless, there is evidence that it can cause contact sensitization. Positive reactivity to sesquiterpene lactones, the allergenic chemicals found in Asteraceae plants, have been shown in patch test investigations. These reactions are more common in those who have a history of ragweed, chrysanthemum, or marigold allergies (Reider et al. 2001; Paulsen 2002).

The inner leaf latex, which contains anthraquinones like aloin, is a recognized irritant and can result in allergic contact dermatitis, including instances of widespread dermatitis after application on stasis ulcers. In contrast, aloe vera (*Aloe barbadensis*) gel is notably non-toxic topically. The latex has known laxative properties when taken orally, and in excessive amounts, it may be nephrotoxic (Morrow et al. 1980; Boudreau and Beland 2006). Calendula (*Calendula officinalis*) has a very minimal potential for irritation and a good safety profile. Its non-irritating and non-sensitizing properties when applied to both intact and injured human skin have been repeatedly proven by repeated Insult Patch Testing (RIPT) investigations, which are the benchmark for assessing cosmetic chemicals (Duran et al. 2005; Parente et al. 2012).

The primary toxicity concerns for curcumin (*Curcuma longa*) is not irritancy but its staining potential on skin and fabrics. Systemic toxicity is exceptionally low, even at high doses. A comprehensive review concluded that doses up to 8 g/day for months were well-tolerated in human clinical trials, with only mild gastrointestinal upset reported in some subjects (Gupta et al. 2013). In contrast, licorice (*Glycyrrhiza glabra*) has significant and well-quantified systemic toxicity. The active constituent, glycyrrhizic acid, inhibits 11β-hydroxysteroid dehydrogenase type 2, leading to pseudoaldosteronism. Studies have established that chronic consumption of as little as 100 mg/day of glycyrrhizic acid can cause a rise in blood pressure and a drop in potassium levels. The European Food Safety Authority (EFSA) and others have set an acceptable daily intake (ADI) of 100 mg/day to prevent these effects (Isbrucker and Burdock 2006; Omar et al. 2012).

The essential oils of lavender (*Lavandula angustifolia*) and peppermint (*Mentha piperita*) are potent and require careful dilution. Lavender oil, while often used for its calming effects, has been identified as a frequent cause of allergic contact dermatitis in aromatherapy patients. Patch testing has confirmed linalool and limonene, especially when oxidized, as the primary allergens (Cavanagh and Wilkinson 2002; Hagvall et al. 2008). Peppermint oil is a strong irritant and can cause sensitization. Its key component, menthol, can cause burning sensations, urticaria, and erythema at high concentrations (> 16%), but is generally safe at low concentrations (1–5%) in topical formulations. A significant number of cases of perioral dermatitis and contact cheilitis have been linked to peppermint oil in lip balms and toothpastes (Ale et al. 2002; Tran et al. 2010).

Recent scientific advances have elucidated the mechanisms and optimized the delivery of natural antipruritics. A primary research focus involves overcoming the poor bioavailability of curcumin through advanced encapsulation techniques. Studies demonstrate that nanoparticle, liposome, and phospholipid complex formulations significantly enhance cutaneous penetration and retention, leading to superior anti-inflammatory and antipruritic efficacy in murine models of allergic contact dermatitis compared to native curcumin (Marques et al. 2023; Liakopoulou et al. 2025).

Investigation into Aloe Vera has expanded beyond its anti-inflammatory properties to include its microbiome-modulating effects. Aloe vera polysaccharides, particularly acemannan, are now recognized for alleviating pruritus by promoting skin barrier integrity and preventing cutaneous dysbiosis, which can exacerbate inflammatory conditions (Swaraj 2024; Bolatkyzy et al. 2025).

Pharmacological research on Peppermint has refined the understanding of menthol’s mechanism. Its antipruritic action is not solely mediated by TRPM8 receptor activation but also involves inhibition of the pro-inflammatory and pruritic TRPA1 channel, indicating a dual pathway for itch suppression (Li et al. 2022). Similarly, studies on Lavender oil confirm that linalool modulates microglial activity, suggesting a central mechanism for reducing neuroinflammation associated with chronic pruritus (Singh and Mishra 2024).

Safety profiles are being re-evaluated with sophisticated models. Patch testing continues to monitor the low but non-negligible sensitization potential of chamomile and calendula, particularly in individuals with compositae allergies (Bingham et al. 2019; Javed et al. 2024). A systematic review concluded that topical licorice extract (glycyrrhetinic acid ≤ 2%) is exceptionally well-tolerated with no reported irritancy or sensitization, positioning it as a safe alternative to low-potency corticosteroids; however, warnings against oral ingestion due to pseudoaldosteronism risk remain (Pastorino et al. 2018b).

## Conclusions

Itch, or pruritus, is a distressing sensation that can significantly impair quality of life. Recent research has highlighted various natural compounds with antipruritic properties, offering potential therapeutic avenues. Chamomile (*Matricaria chamomilla*), aloe vera (*Aloe barbadensis*), calendula (*Calendula officinalis*), curcumin (*Curcuma longa*), lavender (*Lavandula angustifolia*), licorice (*Glycyrrhiza glabra*), and peppermint (*Mentha piperita*) documented as promising antipruritic candidates. Natural antipruritic agents offer a multifaceted approach to itch relief through anti-inflammatory, antihistaminic, and neural modulatory mechanisms. Their integration into clinical practice holds promise for addressing chronic pruritus, particularly in cases resistant to conventional therapies. Despite the encouraging results from both preclinical and clinical studies, challenges persist regarding the standardization of dosages and formulations. The review underscores the need for additional clinical trials to validate the efficacy and safety of these herbal remedies, advocating for their incorporation into complementary palliative care strategies. This holistic approach aims to address both the physical discomfort and emotional distress caused by chronic pruritus, ultimately improving patient well-being. Incorporating these medicinal plants into palliative care necessitates a collaborative approach that merges traditional herbal knowledge with contemporary scientific research. By addressing existing research gaps and establishing evidence-based guidelines, these botanical remedies could become a viable, accessible, and culturally appropriate option for alleviating pruritus while improving the overall comfort of patients in palliative settings. This strategy not only provides relief from challenging symptoms but also aligns with the principles of holistic care by considering both physical and emotional health.

## Data Availability

No datasets were generated or analysed during the current study.
